# Intersegmental coupling and recovery from perturbations in freely running cockroaches

**DOI:** 10.1242/jeb.112805

**Published:** 2015-01-15

**Authors:** Einat Couzin-Fuchs, Tim Kiemel, Omer Gal, Amir Ayali, Philip Holmes

**Affiliations:** 1 Department of Mechanical and Aerospace Engineering, Princeton University, NJ 08544, USA; 2 Department of Zoology, Tel Aviv University, Tel Aviv 6997801, Israel; 3 Department of Kinesiology, University of Maryland, College Park, MD 20742, USA; 4 Sagol School of Neuroscience, Tel Aviv University, Tel Aviv 6997801, Israel; 5 Program in Applied and Computational Mathematics and Princeton Neuroscience Institute, Princeton University, NJ 08544, USA

**Keywords:** Central pattern generator, Intersegmental coordination, Locomotion, Leg perturbation, Phase oscillators

## Abstract

Cockroaches are remarkably stable runners, exhibiting rapid recovery from external perturbations. To uncover the mechanisms behind this important behavioral trait, we recorded leg kinematics of freely running animals in both undisturbed and perturbed trials. Functional coupling underlying inter-leg coordination was monitored before and during localized perturbations, which were applied to single legs via magnetic impulses. The resulting transient effects on all legs and the recovery times to normal pre-perturbation kinematics were studied. We estimated coupling architecture and strength by fitting experimental data to a six-leg-unit phase oscillator model. Using maximum-likelihood techniques, we found that a network with nearest-neighbor inter-leg coupling best fitted the data and that, although coupling strengths vary among preparations, the overall inputs entering each leg are approximately balanced and consistent. Simulations of models with different coupling strengths encountering perturbations suggest that the coupling schemes estimated from our experiments allow animals relatively fast and uniform recoveries from perturbations.

## INTRODUCTION

Cockroaches are renowned for their ability to maintain dynamic stability when running over uneven terrain. Their rapid recovery from unexpected perturbations results from both passive mechanical properties of their musculoskeletal structures and interactions among central and local sensorimotor circuits controlling legs, and hence body dynamics (Jindrich and Full, 2002; Dudek and Full, 2006; Dudek and Full, 2007; Sponberg and Full, 2008; Sponberg et al., 2011a; Sponberg et al., 2011b). These interactions collectively contribute to produce robust coordinated gaits with the flexibility to dynamically adapt leg movement when encountering disturbances. A critical challenge is to discover and understand the inter-leg coupling structure and strengths responsible for this behavior.

Inter-leg coordination during locomotion may be achieved by connections between central pattern generator (CPG) networks controlling each leg, by inter-leg afferent signals, and by mechanical coupling among legs during stance. Inter-leg couplings are likely to differ among species, and may depend on locomotive context (Dürr et al., 2004; Dürr, 2005). Experimental studies of crustaceans and stick insects suggest that each limb has its own inherent stepping rhythm and is only weakly coupled to neighboring legs (Chasserat and Clarac, 1980; Büschges, 2005; Ludwar et al., 2005; Borgmann et al., 2007; Borgmann et al., 2009). This is supported by behavioral observations that suggest sets of reflex-based rules by which information about leg states and positions passes to neighboring legs to time successive steps (Cruse, 1990). In contrast, gait coordination in fast-moving cockroaches is expected to rely as much or more on pre-programmed patterns transmitted from the CPG in a feed-forward manner, than on sensory feedback (Wilson, 1966; Pearson, 1972; for a review, see Ayali et al., 2015).

This was previously investigated by recording from motoneurons in deafferented cockroach preparations (Fuchs et al., 2011; Fuchs et al., 2012), showing that intersegmental coupling among thoracic hemisegments can produce the animal's predominant double tripod gait (Delcomyn, 1971; Pearson and Iles, 1973) and that, while contralateral coupling is symmetric, ipsilateral coupling from rostral units is stronger than from caudal units (Fuchs et al., 2011). Moreover, sensory information from a single stepping leg can reinforce central coupling to achieve tighter coordination (Fuchs et al., 2012). Similar segment-specific effects of sensory feedback on centrally generated motor patterns were observed in semi-intact stick insect preparations (Borgmann et al., 2007, 2009), and phase-response curves (PRCs) that quantify information transfer among hemisegments have been measured in both species (Borgmann et al., 2009; Fuchs et al., 2012).

Mathematical models of hexapedal locomotion have also shown that CPG-driven feed-forward architectures can produce stable tripod gaits (Collins and Stewart, 1993; Ghigliazza and Holmes, 2004). Equipped with biophysically realistic muscles and leg geometries, the models run stably and recover from substantial perturbations (Kukillaya and Holmes, 2009; Kukillaya et al., 2009). The CPG model of Ghigliazza and Holmes (Ghigliazza and Holmes, 2004) couples the hemisegmental oscillators in a manner that preserves equal inputs to all six units. Specifically, because middle legs receive ipsilateral inputs from both posterior and anterior directions, ipsilateral connections entering them were set to half the strength of all other ipsi- and contra-lateral couplings. Interestingly, a similar ratio was found in coupling between hemisegments innervating the middle and hind legs in deafferented preparations (Fuchs et al., 2011). It was, however, unknown whether similar coupling exists in intact running over a range of speeds, or how principles observed at the neuronal level are expressed in behaving animals.

In this paper, we use experiments, mathematical models and simulations to investigate inter-leg coupling structures, gait maintenance and recovery from perturbations in freely-running, intact cockroaches, to complement the deafferented preparations described above. We hypothesize that PRCs for intact animals will be similar to those for the preparations of Fuchs et al. (Fuchs et al., 2011; Fuchs et al., 2012), but that the influence of sensory feedback will decrease with running speed.

We apply localized magnetic perturbations to single legs and monitor the resulting inter-leg phase differences, and use data from unperturbed trials to estimate coupling strengths and other model parameters. Phase oscillator models that describe leg coordination via interactions among six coupled units play a key role in our analyses. They suggest a thoracic network with nearest-neighbor coupling that maintain approximately balanced inputs to each unit, especially at higher speeds. Our methods may be useful in studying other rhythmic processes, and could assist engineers in designing legged robots that provide stable coordination and gait flexibility (Ferrell, 1995; Beer et al., 1997; Quinn et al., 2001; Holmes et al., 2006; Haynes et al., 2012).

## RESULTS

We use both data- and model-based methods, as described in the Materials and methods. The mathematical model comprises six phase oscillators that quantify each leg's state via its progress through the stance–swing cycle. The model is essential to our data analyses and in generating simulated data for comparison with experiments. We employ the following conventions: left and right legs are named L1, L2, L3 and R1, R2, R3, with 1, 2, 3 respectively denoting pro-, meso- and meta-thoracic hemisegments. Numerical indices for the leg phases θ*_i_* in the oscillator model are R1=1, R2=2, R3=3, L1=4, L2=5, L3=6 (see below).

### Magnetic perturbation experiments

**Fig. 1.**
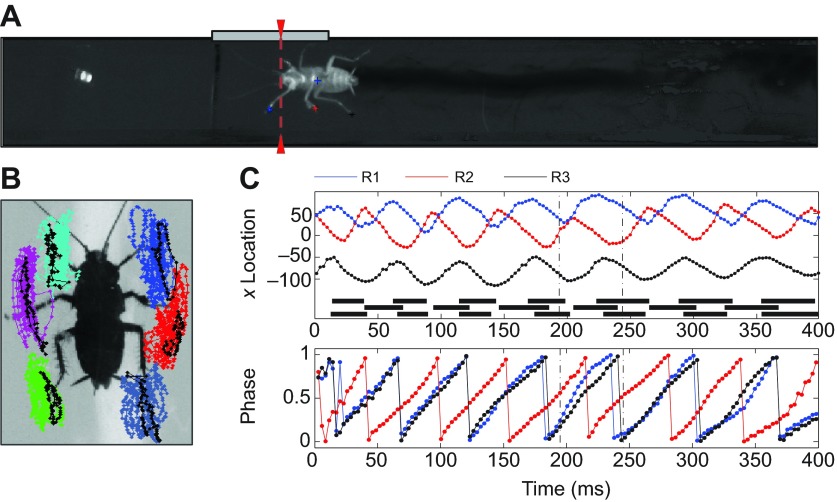
**Experimental set-up and leg kinematics.** (A) The experimental tunnel viewed from below after image processing to reduce background noise. Helmholtz coil location is shown by a gray bar and the optical sensor beam by red dashes. LED at left indicates an active magnetic field in this frame. (B) Locations of tarsi relative to body centroid before (colors) and during (black) magnetic perturbation applied to the right middle leg (R2). (C) Top panel shows longitudinal (*x*)-locations of right tarsi relative to body centroid; black bars indicate stance periods, dashed lines show perturbation duration. Bottom panel shows corresponding phase values.

To investigate recovery from localized perturbations, we applied brief impulses to a micromagnet (mass, 0.0118 g) attached to the tibia of a single front, middle or hind leg as cockroaches (*Periplaneta americana*) passed a Helmholtz coil while running freely along a tunnel (Fig. 1A). Fig. 1 also shows tarsi trajectories relative to the body center of mass (Fig. 1B) during a typical trial with a magnet on the middle right leg. Vertical dashed lines (Fig. 1C, top) denote the perturbation duration *T*_mag_=50 ms. Leg phases (Fig. 1C, bottom) were computed via Hilbert transforms of high speed camera images as described below. Here, tarsi trajectories during the perturbed cycle show step shortening in the middle right leg (Fig. 1B, black; cf. unperturbed trajectories in red). Effects depended on leg phase and position at perturbation onset, and varied among trials. Step shortening was typically observed when legs were in anterior positions, while no change or slight lengthening was observed for perturbations applied in posterior positions.

Average running speeds before and after perturbations were 39±14.81 and 37.95±16.06 cm s^−1^; speeds reduced during the perturbed cycle by −1.55±5.75 cm s^−1^. Corresponding means and s.d. of body yaw angles with respect to the tunnel were −0.058±0.120 deg and −0.063±0.118 deg. Paired *t*-tests showed no significant speed or yaw changes because of perturbations, and runs without coil activation confirmed that the magnet alone had no effect on kinematics. See Appendix for further details.

### Effective phase-response curves

**Fig. 2.**
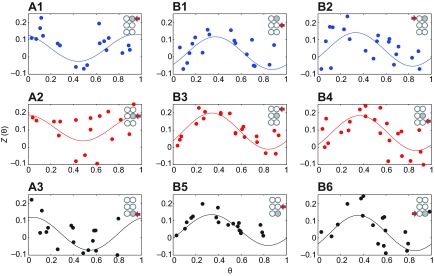
**Effective phase-response curves versus phase of stimulation onset at the perturbed leg.** (A1–A3) Self-perturbations in legs R1, R2 and R3. (B1–B6) Responses in neighboring legs to perturbations in R1, R2 and R3 (top to bottom). Schematics show where perturbation was applied (gray hemisegment) and response was measured (red arrow). EffPRCs were fitted to data (dots) using first-order Fourier series (solid curves).

As noted above, leg responses depend upon phases at which perturbations arrive. To study this, we measured effective phase-response curves (effPRCs) at each perturbed leg and at all ipsi- and contralateral sites neighboring it. These characterize the sensitivity of a periodic process to inputs at different phases on its cycle. Examples appear in Fig. 2, illustrating that perturbations have relatively small but consistent phase-dependent effects, e.g. *Z*(θ)=+0.1 denotes a 10% decrease in cycle duration, compared with values as high as 0.6 for steps imposed on a single intact leg in a partially deafferented preparation (Fuchs et al., 2012; Fig. 2). EffPRCs for self-perturbations peak at θ=0, indicating maximal responsiveness around touchdown, and have smallest and possibly negative values at θ=0.5, close to lift-off (Fig. 2A1–A3). See Appendix and supplementary material Fig. S1 for further details.

In contrast, effPRCs for neighboring perturbations (Fig. 2B1–B6) peak during stance at θ≈0.35, and show less effect during swing (θ≈0.5–1). Nonlinear regression analysis comparing the first-order Fourier series (details in the Materials and methods), revealed no statistically significant differences between all pairs of effPRCs for neighboring perturbations, suggesting that the effect of perturbations spreads in a relatively uniform way, producing similar responses in all neighboring legs. EffPRCs for self-perturbations also do not differ significantly among themselves, but they are significantly different from effPRCs for neighboring legs (*P*<0.05 between all A versus B curves in Fig. 2).

### Recovery from perturbations

**Fig. 3.**
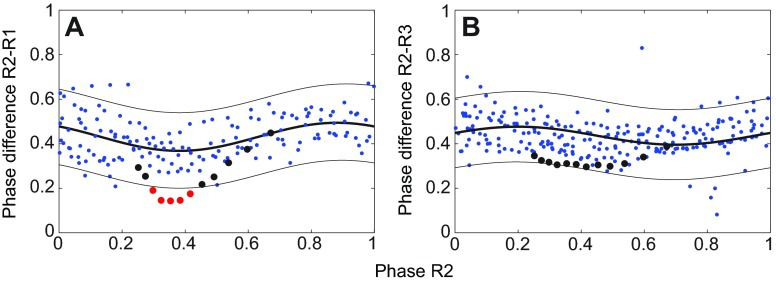
**Phase differences between leg pairs before and during perturbations.** Phase differences before (small blue points) and during (black and red dots) a perturbation in R2 are plotted versus phase of R2 for a single animal. First-order Fourier series fits and estimated 95% prediction intervals for pre-perturbation phase differences (thick and thin black curves). Data for neighboring ipsilateral pairs R2-R1 (A) and R2-R3 (B) show examples in which phase differences deviated (red dots) and did not deviate (black dots) from prediction intervals.

As described in the Materials and methods, we characterized unperturbed activity by analyzing phase differences between all leg

pairs during running to establish 95% prediction intervals. Tripod-like gaits were observed in all trials (Fig. 1C), although interleg phase differences varied though the cycle. Fig. 3 shows two examples that include data during magnetic perturbations, with red and black dots indicating significant and insignificant deviations from unperturbed running. The systematic sinusoidal fluctuations may be due to imperfect phase estimates from the Hilbert transform method; similar results were also obtained using the Phaser algorithm (Revzen and Guckenheimer, 2008).

**Fig. 4.**
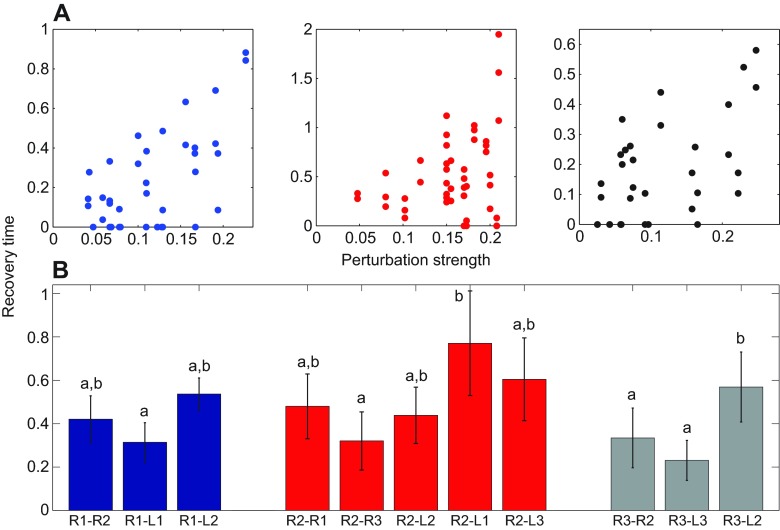
**Recovery from perturbations.** (A) Recovery times in cycle fractions versus self-perturbation strengths |Z*_i_*(θ*_ik_*)| for perturbations at R1, R2 and R3, measured at all ipsi- and contralateral legs neighboring the perturbation site. See text for details. (B) Mean (± s.d.) of normalized recovery times for each neighboring leg pair after perturbations in R1 (blue), R2 (red) and R3 (gray). Two-way ANOVA analysis classified recovery values in groups a, a,b and b; values in a differ significantly from those in b, but those in a,b do not differ significantly from a or b.

Fig. 4A illustrates the dependence of recovery times on self-perturbation strength. These strengths were estimated using effPRCs (details in the Appendix) evaluated at the perturbed leg, and recovery times determined as fractions of cycles in which phase differences deviate from 95% prediction intervals of unperturbed data (red points in Fig. 3A). Recovery times for different trials and a given leg are shown in each panel: the approximately linear envelope is consistent with using perturbation strength to normalize recovery times.

Fig. 4B shows normalized mean recovery times *n_i,j_* (Eqn 7) for all neighboring legs following perturbations in R1, R2 and R3. We confirmed bilateral symmetry and generally assume it throughout (a specific example is cited in the following Results section). We therefore regard this data as describing analogous effects of perturbations in the left legs. All leg pairs recover within one cycle, the fastest being the front and hind contralateral pairs R1-L1, R3-L3 and R2-R3. Statistically significant differences were not found between any adjacent ascending, descending or contralateral pairs (two-way ANOVA), but diagonal pairs (R1-L2, R2-L1, R2-L3 and R3-L2) exhibit longer recovery times than adjacent pairs, although only those of R2-L1 and R3-L2 are significantly different. Assuming that stronger coupling between a leg pair results in faster recovery, this finding suggests an architecture with nearest-neighbor connections rather than one in which the units of each tripod are connected diagonally. We describe further support for this below.

### Intersegmental coupling

**Fig. 5.**
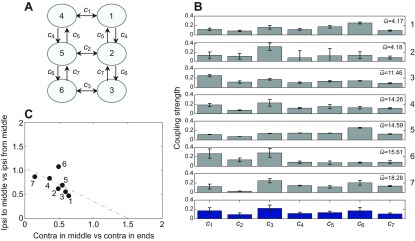
**Coupling strength studies.** (A) The connection architecture and coupling strengths *c*_1_,…,*c*_7_ between hemisegmental units. (B) Estimated coupling strengths for seven preparations with average stepping frequencies 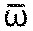 indicated. Bars show s.e. of parameter estimates (see the Materials and methods for details). To aid comparison, coupling values of each preparation were normalized so that strengths sum to 1; non-normalized values varied among preparations with mean 76.1±51.4. Bottom row shows means over all preparations and error bars note s.d. of the seven preparations. (C) Ratios between ipsilateral couplings entering and leaving middle units [(*c*_4_+*c*_7_)/(*c*_5_+*c*_6_)] versus ratios between contralateral coupling in middle and end units [2*c*_2_/(*c*_1_+*c*_3_)] for each preparation, numbered as in B; linear regression fit shown dashed.

Estimates of inter-unit coupling strengths were obtained from unperturbed runs, as described in the Materials and methods. Touchdown sequences from seven additional intact preparations,

each traversing the tunnel 8–12 times at a relatively constant speed, were used in the analysis. We chose a nearest-neighbor coupling architecture with only ipsi- and contralateral connections (see Fig. 5A). Preliminary studies of architectures that also included diagonal connections showed that fit qualities did not justify the additional parameters. (See below and Appendix for details;

diagonal connections have been postulated, although we are unaware of physiological evidence for them). Allowing different coupling strengths between each pair of units in both directions, there are potentially 14 values to estimate, along with the other 13 parameters in the linearized stochastic oscillator model.

To investigate how many independent coupling parameters suffice to capture the dynamics of touchdown sequences, we computed maximum-likelihood estimates for models with increasing degrees of bilateral and rostro-caudal symmetry, using Akaike and Bayes information criteria (Akaike, 1974; Akaike, 1981). We started with 14 coupling strengths (as above), and then considered 11 (assuming R–L symmetry in all contralateral connections), nine (partial bilateral symmetry), seven (full bilateral symmetry), six (full bilateral and partial rostro-caudal symmetry), four (full bilateral and rostro-caudal symmetry), three, two and one. Details of specific constraints for this comparative study are given in the Appendix and supplementary material Table S1.

We found that seven different strengths were sufficient and necessary to produce acceptable touchdown sequences, suggesting that deviations from bilateral symmetry are negligible, in agreement with our earlier study of deafferented preparations (Fuchs et al., 2011). Henceforth, we consider only the bilaterally symmetric architecture of Fig. 5A, with contralateral coupling strengths *c*_1_, *c*_2_, *c*_3_ and ipsilateral strengths *c*_4_, *c*_5_, *c*_6_, *c*_7_. Fig. 5B shows the resulting values for the seven preparations, along with values averaged over all preparations. Contralateral coupling between the middle units (*c*_2_) is weaker than that between the hind units (*c*_3_) for all preparations (*P*<0.05, two-way ANOVA), and weaker than that between the front units (*c*_1_) in most preparations.

Despite substantial differences in absolute coupling strengths among the preparations, we also found a consistent relationship between ratios of coupling strengths entering and leaving the middle units (absolute values are given in supplementary material Tables S2 and S3). Fig. 5C plots ratios between ipsilateral couplings entering and leaving the middle units (*c*_4_+*c*_7_)/(*c*_5_+*c*_6_), versus ratios between contralateral coupling in the middle and end units 2*c*_2_/(*c*_1_+*c*_3_) for each preparation. With one exception (the 15.6 Hz case), all preparations lie close to a regression line in this two-dimensional space, indicating a trade-off between contralateral and ipsilateral coupling ratios. This may have implications for stability and control of locomotion, as discussed below. We also note that absolute coupling strengths correlate with intrinsic noise levels estimated for each preparation, and that higher speed runners generally have larger strengths and higher noise levels (although the 14.59 Hz preparation is an exception) (see Appendix and supplementary material Fig. S2).

### Relationship between coupling strengths and recovery times

**Fig. 6.**
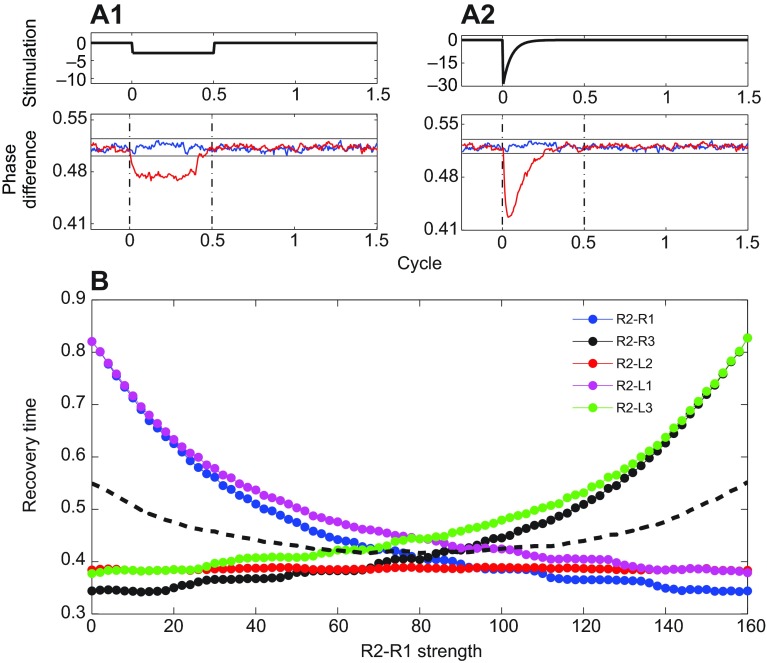
**Simulations of the phase oscillator model.** (A1,A2) Phase differences between R1 and R2 (bottom panels, red) in response to constant and exponentially decaying perturbations of half-cycle duration at R2 (top). Unperturbed data shown in blue with s.e. (black lines); dashed vertical lines indicate cycle duration. (B) Recovery times after perturbation of type A2 at R2 for different coupling strengths from R2 to R1 and R3. Black dashed curve shows average over all leg pairs. Here, 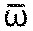
=10 Hz and *c_j_*=80 for all coupling strengths except *c*_5_ and *c*_6_;*c*_5_ varies from 0 to 160 with *c*_6_=160−*c*_5_.

We used the nonlinear oscillator model as described in the Materials and methods with stochastic terms to simulate the effects of perturbations. A rectangular step and an exponentially decaying function were considered as descriptions of the magnetic perturbation (Eqn 10). We found that the latter produced phase changes in better agreement with the data, as shown in Fig. 6A1,A2, and we used it in all computations reported here.

To investigate how nonuniform coupling affects recovery, we initially set all *c_j_*=80, frequency at 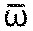
=10, and noise levels at σ*_i_*=1.0, representative of non-normalized values found above (see supplementary material Tables S2 and S3). Since recovery times are expressed in cycle fractions, only the relative magnitudes of these parameters influence the results. We then varied the outgoing connections *c*_5_ and *c*_6_ from R2 to R1 and to R3 (cf. Fig. 5A), maintaining bilateral symmetry but breaking rostro-caudal symmetry by setting *c*_6_=160−*c*_5_. Perturbations of duration *T*_mag_=50 ms were applied to R2 while recovery times for all other legs were computed as *c*_5_ was raised from 0 to 160 and *c*_6_ decreased accordingly.

Fig. 6B shows that the maximally symmetric system, with *c*_5_=*c*_6_=80, has the smallest spread and fastest average of recovery

**Fig. 7.**
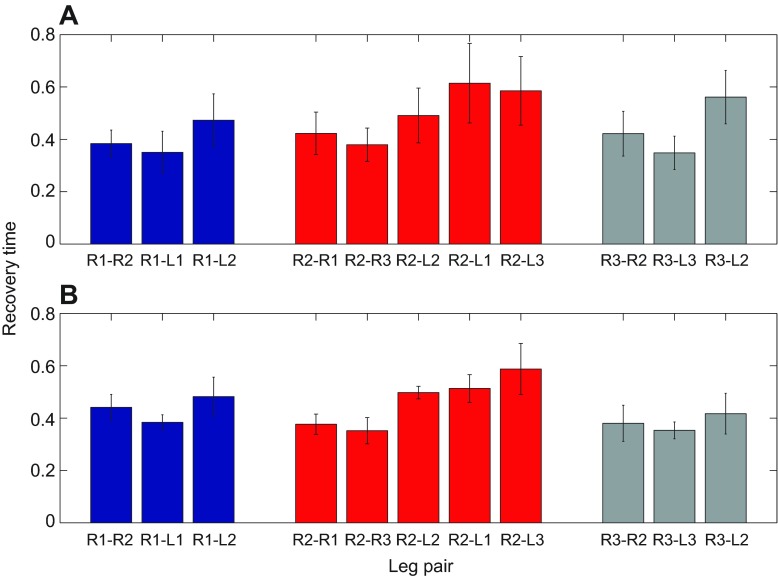
**Recovery times for the phase oscillator model.** (A) Mean recovery times in cycle fractions for the model with parameters fitted separately to each preparation. (B) Recovery times for model simulations using a single set of averaged parameters, as in Fig. 5B (bottom row). Error bars denote s.d. over 50 runs. In both panels, blue, red and gray bars indicate perturbations in R1, R2 and R3, as in Fig. 4B.

times (black dashed curve). Also, in agreement with the intuition that strong coupling promotes rapid recovery, phase differences between R2's nearest neighbors R1, R3 and L2 recover more quickly than those between its diagonal neighbors L1 and L3, two connections distant from R2. Increasing R2-R1 coupling *c*_5_ reduces recovery times of R1, L1 and L2, but increases them for R3 and L3. Reducing *c*_5_ reverses this effect, showing that symmetry breaking has a cost in that recovery rates of different leg pairs spread over wider ranges.

We then ran seven versions of the model with parameters estimated from the experimental runs described in the Intersegmental coupling section above, using non-normalized values of coupling strengths *c_j_* along with preferred phase differences ψ*_ij_*, frequency 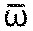, and noise levels σ*_i_* for each preparation (cf. Fig. 5B). Each version was simulated 50 times to obtain leg-pair recovery times, and these were then averaged over the models to produce Fig. 7A. We also simulated an ‘average preparation’ by taking the non-normalized parameter values averaged over all seven preparations (cf. bottom row of Fig. 5B). These results appear in Fig. 7B.

Neither these simulations nor the recovery from pertubations experiments reveal significant differences in recovery times of legs located anteriorly versus posteriorly to the perturbed leg. However, in accord with weaker contralateral coupling between the middle legs, we do observe slower recoveries from perturbations in those segments compared with contralateral front and hind pairs. Interestingly, differences among experimental recovery times are more similar to those obtained from the parameters of each preparation separately than to those of the averaged preparation (cf. Fig. 4B with Fig. 7A vs 7B). This is consistent with the variability in coupling strengths among animals of Fig. 5B.

### Recovery performance, speed dependence and distance from balance

**Fig. 8.**
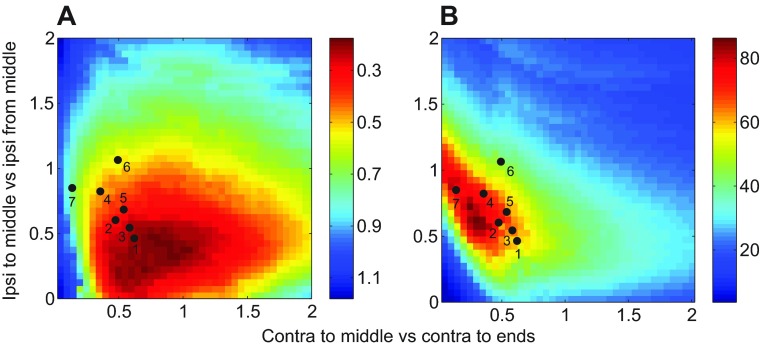
**Recovery performances of different coupling schemes.** Mean recovery times averaged over all leg pairs (A), and recovery homogeneity (B) are represented in the plane spanned by ratios of contralateral and ipsilateral coupling strengths 2*c*_2_/(*c*_1_+*c*_3_) and (*c*_4_+*c*_7_)/(*c*_5_+*c*_6_) (cf. Fig. 5C). Color scales represent recovery time and homogeneity; coupling schemes of experimental preparations are shown as black dots. Recovery homogeneity is the inverse of variance among recovery times in neighboring legs. Recovery times were computed as described in the text.

In Fig. 5C we characterized the different preparations in terms of ratios of ipsilateral and contralateral coupling strengths: (*c*_4_+*c*_7_)/(*c*_5_+*c*_6_) and 2*c*_2_/(*c*_1_+*c*_3_). Fig. 8 displays the same experimental data (black dots) superimposed on color maps quantifying recovery times (A) and recovery homogeneity (B) over the parameter space. Recovery times were generated by simulating models with different coupling values *c_j_* and averaging over all leg pairs, as in Fig. 6, while keeping the sum of coupling strengths 
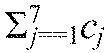
 fixed.

Specifically, we set 
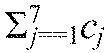
/7=80, 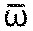
=10 and ψ*_ij_*=0 and 0.5, consistent with a tripod gait, and sampled the resulting 6-dimensional

**Fig. 9.**
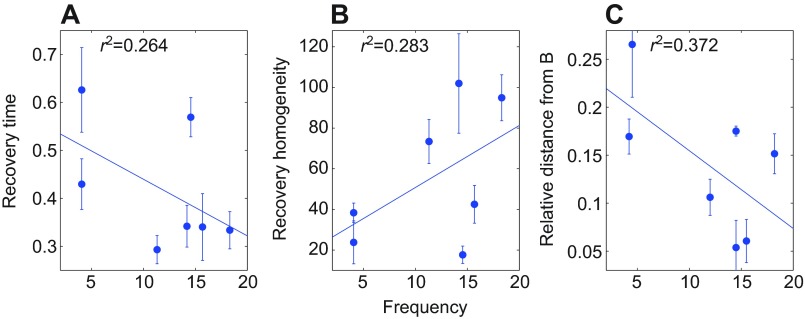
**Are different connectivity schemes exhibited at different stepping frequencies?** Dependence of recovery times (A), recovery homogeneities (B) and relative distances from the balanced subspace (C). Relative distances are described in the text. Error bars in A and B are computed as described in the text; in C they are s.e. of coupling strength estimates from Fig. 5B.

coupling space by choosing ratios of *c*_1_/*c*_3_, *c*_4_/*c*_7_ and *c*_5_/*c*_6_ as 0.5, 1 or 2. We averaged over the 3^3^=27 parameter sets to obtain a mean recovery time for each point in a grid on the [2*c*_2_/(*c*_1_+*c*_3_), (*c*_4_+*c*_7_)/(*c*_5_+*c*_6_)] plane. Recovery homogeneity was defined as the inverse of variance among recovery times in all neighboring legs following each perturbation. Note that six of the seven preparations lie within or close to the region of maximal homogeneity (Fig. 8B) and four of them are close to the minimum recovery time (Fig. 8A).

We next asked whether the recovery properties displayed in Fig. 8 are related to stepping frequency, and hence to running speed. Fig. 9A reveals a weak negative correlation with mean recovery times: with one exception (the 14.59 Hz case), higher frequency preparations have shorter recovery times. There is also a weak positive correlation with recovery homogeneity (Fig. 9B). Although differences between slow and fast preparations are not statistically significant, these results indicate that faster animals tend to recover in a smaller fraction of their step cycles and more uniformly than slower ones. Mean values and error bars in Fig. 9A,B were computed by simulating 100 models with parameters drawn from Gaussian distributions with mean values and variances estimated from the data (see supplementary material Tables S2 and S3).

To discuss Fig. 9C, we must first describe the notion of balanced inputs. The middle units L2, R2 in Fig. 5A each have three entering connections; all other units have two. For the net input strengths to all six units to be equal, the condition:
(1)
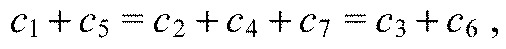

must hold, defining a balanced subspace *B* in the 7-dimensional space of coupling strengths. In cockroach CPG modeling, before connection strength data became available, a special case of balance was assumed: *c*_j_=*c* for *j*=1,2,3,5,6 and *c*_4_=*c*_7_=*c*/2 (Ghigliazza and Holmes, 2004). This corresponds to the point (1,0.5) on Fig. 5C, close to the regression line, suggesting that we investigate the more general case of Eqn 1.

To determine how close the seven preparations are to balance, we computed Euclidean distances of normalized coupling vectors shown in Fig. 5B from *B*, as described in the Appendix. The results, shown in Fig. 9C, confirm that all seven are relatively close to *B* in comparison with their spread parallel to *B*. Here we plot relative distances from *B*, i.e. ratios of the distance of each coupling vector *c*=(*c*_1_,…,*c*_7_) from *B* to the component of its magnitude parallel to *B*. These distances are all below 0.27, and Fig. 9C also shows that the faster preparations are significantly closer to balanced than slower ones (*P*<0.05). Further work is needed to investigate these patterns during different locomotive tasks and determine how animals modulate coupling strengths during locomotion in natural conditions.

## DISCUSSION

In this paper, we present a stochastic six-oscillator model for hexapedal locomotion and fit its parameters using data from freely running cockroaches (*Periplaneta americana*). The model derives from previous biophysically based studies (Kukillaya and Holmes, 2009; Kukillaya et al., 2009; Proctor et al., 2010) and represents each hemisegmental leg unit by a single phase angle, tracking progress through the stepping cycle (Fig. 1). It is simple enough to produce estimates of inter-leg coupling architectures and strengths, preferred phase differences between leg pairs and other parameters. We use sinusoidal coupling functions to describe each leg units' phase sensitivity to inputs from other legs. Such functions are predicted by analyses of bursting neuron CPG models (Ghigliazza and Holmes, 2004) and further justified by measured phase-response curves that quantify how inputs at different phases are transferred to neighboring legs (Fig. 2).

### Recovery times, network architecture and coupling strengths

We computed recovery times for transient perturbations applied at single legs, finding that the tripod gait resumes in less than a stride in almost all cases (Figs 3, 4). Similar recoveries, shorter than a stance or swing phase, were previously observed in isolated, impulsively perturbed cockroach legs and attributed to viscoelastic exoskeletal joints and muscle properties (Dudek and Full, 2006; Dudek and Full, 2007). Following lateral perturbations Revzen et al. (Revzen et al., 2013) observed a delayed decrease in stride frequency from kinematic leg phases and muscle activity, suggesting that recovery begins with self-stabilizing mechanical feedback followed by neural feedback that changes the CPG frequency. We saw slight, but insignificant, speed reductions.

As in earlier studies, we used intrinsic cycle-to-cycle variability in long stepping sequences to fit parameters and a maximum-likelihood method to optimize them (Kiemel and Cohen, 1998; Kiemel et al., 2003; Fuchs et al., 2011), simplifying the procedure by fitting a linearized model near a phase-locked solution (cf. Roth et al., 2014). Our parameter estimates confirm approximate bilateral symmetry and favor nearest-neighbor coupling. Coupling strengths vary among different preparations, but exhibit patterns that yield rapid and uniform recoveries (Figs 5, 6 and 7).

Interleg coupling strengths in stick insects were previously estimated from the precision of swing and stance transitions (Dürr, 2005). Stick insects employ varied gaits and exhibit weak contralateral coupling (Graham, 1972; Dürr, 2005), and their inter-leg coordination relies mainly on afferent influences (Borgmann et al., 2012). Central intersegmental coupling is thought to play a stronger role in cockroaches, which approximately maintain double tripod gaits when crossing barriers near the height to which their front tarsi lift (Watson et al., 2002) and traversing obstacles as high as their body–coxa joints (Sponberg and Full, 2008). Despite such differences, similarities in inter-leg coupling in these insects exist, including weak contralateral connections between the middle legs and differing strengths between other leg pairs. Both show strong context dependence, suggesting that coupling strengths may be modulated for different behaviors (Dürr, 2005).

### Possible context- and speed-dependent control

Estimates of coupling strength for our preparations differ in levels of contralateral vs ipsilateral contributions, as expressed in their locations on the regression line of Fig. 5C. Simulations of recovery times and homogeneities over a broad parameter set reveal that most preparations lie close to the minimum recovery time and maximal homogeneity region (Fig. 8A,B). Recovery times and homogeneities are also weakly correlated with leg cycle frequency (Fig. 9A,B), which may also appear in Fig. 4A. There is a significant correlation between frequency and distance from the balanced subspace, indicating that faster animals display greater uniformity in inter-segmental coupling among all legs (Fig. 9C).

### Feedforward and feedback pathways

Our parameter estimates derive from free running and therefore reflect combined effects of central coupling and direct and indirect sensory and mechanical feedback. EffPRCs show differences in receptivity between self- and inter leg perturbations (Fig. 2A vs 2B), suggesting different roles for sensory feedback at different phases of the stepping cycle. Maximal responsiveness to inter-leg perturbations during stance may be due to higher load signals, whereas the perturbed leg itself is mostly affected near touchdown via stretch and position signals. EffPRCs for partially deafferented preparations with a single intact leg also showed maximal responsiveness to step-like perturbations during stance (Fuchs et al., 2012), although, as noted above, their magnitudes are larger than those of Fig. 2. Other similarities between deafferented and intact preparations include bilateral symmetry and stronger coupling from the meta- to meso-thoracic segments in comparison with the other direction.

Fictive locomotion is typically slow [1–2 Hz (Fuchs et al., 2011)], so we cannot compare speed-related differences in intact running, but we expect that the relative contributions of neural, sensory and mechanical factors will vary with behavioral context and speed (Full et al., 2002; Zill et al., 2004; Holmes et al., 2006; Ayali et al., 2015), especially because neuronal properties themselves are likely to change with behavioral conditions (Dickinson et al., 2000; Pfeifer et al., 2007; Roth et al., 2014). For example, genetic manipulations that disrupt sensory pathways in flies show that deprivation of proprioception has a greater effect at lower walking speeds (Mendes et al., 2013). This is consistent with the hypothesis that high-speed coordination relies on central connections and feedforward pathways, but at lower speeds, when the need of precision is greater and dynamic stability weaker, sensory feedback plays a more important role.

Thus, our findings suggest that central coupling is relatively uniform and that lesser uniformity observed in slower preparations may be due to stronger leg- or hemisegmental-specific sensory modalities that are active in slow walking. We hope to address this in future studies, to determine whether it holds across different locomotive tasks, and to test whether, and how, coupling schemes are dynamically modified under more realistic conditions.

## MATERIALS AND METHODS

### Experimental setup and procedures

Experiments were conducted on adult female cockroaches (*Periplaneta americana* Linnaeus 1758) obtained from our colony at Tel-Aviv University. Animals were kept in dark chambers before being released to run along a 600×70×70 mm Plexiglass tunnel, while a camera placed below the tunnel (Prosilica GT2000, AVT, Stadtroda, Germany), captured their motion at 350 fps. An array of LEDs covered with an opaque Plexiglass sheet beneath the tunnel created uniform illumination sufficient for high-speed image capture.

A 138-mm-diameter Helmholtz coil apparatus with N–S poles transverse to the tunnel length was placed on the right side of the tunnel, creating a region of approximately uniform magnetic field across the tunnel along the coil's central axis. Similar coils have been used for controlled perturbations of fruit flies and moths in flight (Ristroph et al., 2010; Dickerson et al., 2014).

To deliver local perturbations on a single cockroach leg, a 1.59 mm (diameter)×0.79 mm, 0.0118 g, grade N52 magnet (K&J Magnetics, Inc., Plumsteadville, PA, USA) was glued to the tibia, oriented to cause a lateral force on entering the magnetic field. We ensured that the magnet's weight had no influence on kinematics, and care was taken to ensure that magnets were placed at the same position and orientation on all animals. We established that insects exhibit no significant deviations from bilateral symmetry, and therefore applied perturbations only to legs on the right side.

An infrared LED bulb opposite a custom-made optical sensor detected objects interfering with the infrared beam as shown in Fig. 1A. Upon detecting an animal passing, the sensor triggered a digital stimulator (Master 8, AMPI Inst. Ltd, Jerusalem, Israel), connected via a solid-state rely to a power generator that delivered precisely timed current pulses to the coils, activating the magnetic field. Peak fields were ≈2×10^−3^ Tesla and currents of 2.5 A and 50 ms duration were used throughout.

In the perturbation experiments, 16 animals traversed the tunnel at relatively constant speeds (<20% variation during each trial). In five of these, the magnet was attached to the tibia of the front right leg, in six, to the middle right and in five, to the hind right. Each animal ran for at least five trials. We tested for speed and path changes following perturbation onset and found no significant effects on body kinematics associated with the perturbations. In addition, seven animals ran through the tunnel without magnetic perturbations, so that the natural variability of their leg kinematics could be used to estimate model parameters.

### Image processing and digitization of tarsi locations

Images were downloaded and stored on a desktop computer as a sequence of TIFF frames with 1024×128 pixel resolution and processed using automatic tracking software developed in Matlab that detects and digitizes locations of all tarsi tips, the body centroid, and body orientation.

Noise was reduced by subtracting a background image calculated by averaging over all frames in each movie sequence. Body position and orientation were determined by detecting the edges of the body contour and finding its axis of symmetry. Tarsi tips were defined as the points of each leg most distal from the silhouette of the body's ellipsoid. This was done manually on the first image of each sequence and subsequently via predicted regions in which each leg could be found relative to the body centroid and tarsi locations on the previous image. During processing, the software allowed monitoring and interactive error correction by manual clicking on correct tarsi positions. Tarsi positions were accurately detected in 90% of cases.

### The phase-oscillator model

To describe and analyze leg dynamics in both free and perturbed running, we model the locomotive system as six coupled phase oscillators (Ghigliazza and Holmes, 2004; Holmes et al., 2006; Kukillaya and Holmes, 2009; Kukillaya et al., 2009; Proctor et al., 2010). We envisage the entire CPG and locomotive apparatus as a dynamical system: a (large) set of ordinary differential equations, containing six subunits describing the thoracic hemisegments and their legs, along with internal neural coupling, mechanical coupling and sensory feedback. Unlike other studies (Kukillaya et al., 2009; Proctor et al., 2010), in which interneurons, motoneurons, muscle complexes and leg dynamics were separately modeled, here, a single phase oscillator represents the components within each hemisegmental unit.

Phase-reduction theory provides a basis for this extreme simplification (Malkin, 1956; Winfree, 2001). If each isolated unit possesses a stable limit cycle (a periodic orbit attracting nearby solutions), then its state can be approximated by the phase θ*_i_*(*t*)ϵ[0,1] describing progress around that cycle. The effect of the *j*-th unit (*j*≠*i*) on θ*_i_*(*t*) is modulated by a phase-response curve (PRC) *Z_i_*(θ*_i_*) that quantifies its sensitivity to inputs at different points on the cycle. *Z_i_*(θ*_i_*) describes the change in phase due to a small delta function impulse applied at θ*_i_* and so is sometimes called the instantaneous PRC (iPRC) (Netoff et al., 2012) [see Chapter 8 in Ermentrout and Terman (Ermentrout and Terman, 2010), Schultheiss et al. (Schultheiss et al., 2012) and Holmes (Holmes, 2014) for applications to neuroscience].

The entire system can therefore be reduced to six phase equations:
(2)
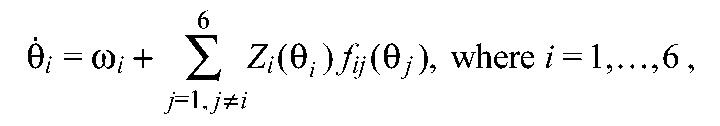

where ω*_i_* is the frequency (Hz) of each uncoupled unit, and the functions *f_ij_* describe couplings among them. If inter-unit coupling is weak relative to the internal dynamics of each unit, it can be proved that Eqn 2 is well approximated by a system in which the right-hand side is averaged over one cycle (Ermentrout and Terman, 2010; Holmes, 2014):
(3)
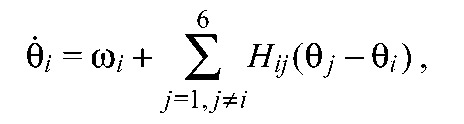

in which the PRCs and coupling architecture are encoded in periodic coupling functions *H_ij_* that depend only on phase differences. Self-interaction terms *H_ii_*(0) are absent because each unit's internal dynamics are described by 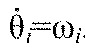.

Generally, coupling functions must be represented by Fourier series, but diverse ion-channel bursting mechanisms have yielded similar PRCs (e.g. Prinz et al., 2003a; Prinz et al., 2003b; Ghigliazza and Holmes, 2004; Prinz et al., 2004) and phase reductions of detailed CPG models (Ghigliazza and Holmes, 2004; Várkonyi et al., 2008) have produced coupling functions that resemble sinusoids with preferred inter-unit phase differences ψ*_ij_*. This supports our explicit choice
(4)
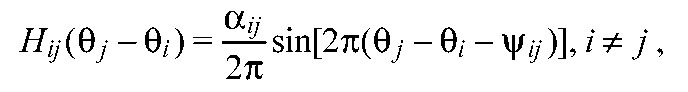

in which α*_ij_* quantifies coupling strength from unit *j* to unit *i*. Similar coupling terms were used previously to model hemi-segmental units in cockroaches (Fuchs et al., 2011) and in lamprey CPGs (Kiemel and Cohen, 1998). Although weak coupling is (presently) necessary for proof of reduction to Eqns 3, 4, the fact that such models can describe not only networks of bursting neurons, but also biomechanical oscillators actuated by nonlinear muscles, has been amply demonstrated [see Proctor and Holmes (Proctor and Holmes, 2010); Proctor et al. (Proctor et al., 2010) for cockroaches].

We now suppose that Eqns 3 have a phase-locked solution in which all oscillators share the same frequency, 
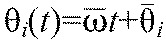
, with 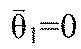 and constant phase differences 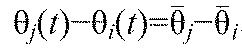, so that 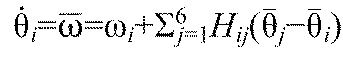. Linearizing Eqns 3 at this solution, we obtain:
(5)
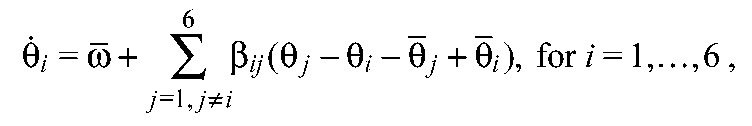

where 
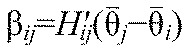
 for *j*≠*i* is the derivative of the function *H_ij_*, evaluated at 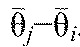. Eqn 5 can be written in vector form 
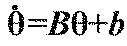
, where **θ**=(θ_1_,…,θ_6_)^T^, and the components *B_ij_*=β*_ij_* for *j*≠*i*, 
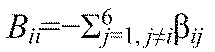
 and 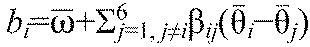. Without loss of generality we can also set 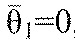, because only phase differences appear in Eqn 5. The Jacobian matrix ***B*** has eigenvector (1,…,1)^T^ with neutral eigenvalue 0, corresponding to perturbations that advance all phases equally. Details of the derivation of Eqn 5 appear in the Appendix.

For Eqn 4, 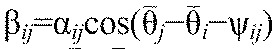 for *j*≠*i*, and therefore β*_ij_*=α*_ij_* if the observed phase differences 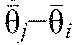 equal the preferred phase differences ψ*_ij_*. Neglecting the small (
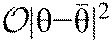
) terms, the linearized system (Eqn 5) will be used to estimate parameters from data obtained in runs without magnetic perturbations. We add noise terms to accommodate variability in the data, and assume that the mean phase differences 
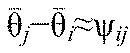
.

### Data analyses

Sequences describing the longitudinal coordinates *x_i_*(*t*) of tarsi positions relative to the body centroid were used to create vectors of phase variables (see Fig. 1B). Each sequence was normalized by subtracting the mean tarsus position and rescaling *x_i_*(*t*) to have standard deviation 1. Normalized leg trajectories were converted into instantaneous phase variables θ*_i_*(*t*) by computing their Hilbert transforms (Huang et al., 1998; Revzen and Guckenheimer, 2008), and scaled to range from 0 to 1, with θ*_i_*=0 when the tarsus is at its anterior extreme position, indicating touchdown (Fig. 1C).

### Effective phase-response curves

Unlike the instantaneous PRCs described above, effPRCs arise from extended perturbations. Similar functions were used to include synaptic dynamics and reversal potentials in Proctor and Holmes (Proctor and Holmes, 2010) and to describe effects of induced leg movements in Fuchs et al. (Fuchs et al., 2012). EffPRCs, like PRCs, describe phase changes as functions of perturbation phase, and may be found from changes in the step cycle length containing the perturbation. We estimated the pre-perturbation period, *T*_mean_, by averaging over the last five cycles preceding perturbation onset. Perturbation phase θ was determined by the interval *S* between the previous touchdown and perturbation onset, and the resulting phase change by the duration *T_p_* of the cycle containing perturbation onset. The dimensionless phase θ and the effPRC *Z*(θ) are then:
(6)
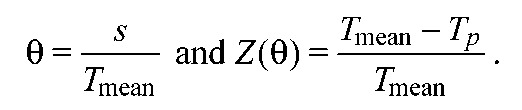



To compare effPRCs at different legs, we fit phase change data from all animals for each leg pair with first order Fourier series, and used F-statistics (Motulsky, 2004) to calculate the likelihood that each two curves are different. Fig. 2 shows examples for self- and neighboring-leg perturbations.

### Recovery from perturbations

We quantified recovery time in terms of stepping cycles required for phase differences between each pair of legs to resume their pre-perturbation range, defined as the 95% prediction interval around a first-order Fourier series that best fit the pre-perturbation data for pairwise phase differences along the stepping cycle. Phase differences deviating from these intervals were designated perturbed and the number (or fraction) of perturbed cycles computed for each trial.

Recovery time depends on perturbation strength, which depends on the magnet's orientation relative to the coil as the insect passes it, and therefore varies from trial to trial. Perturbation strengths for each pair of legs were estimated using effPRCs *Z_i_*(θ*_i,k_*) for self-perturbations of leg *i*, carrying the magnet, and averaged over all trials *k* for the leg pair that yielded perturbed phase differences, to create the normalized mean recovery time for the leg pair *i,j*:
(7)
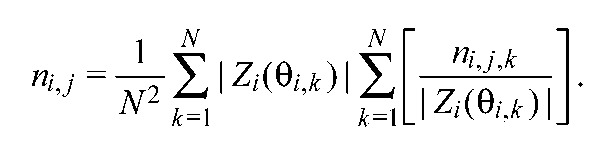



Eqn 7, derived in the Appendix, ensures that recovery times reflect inter-leg connectivity and not responsiveness to perturbations at specific phases. Expressing recovery times in cycles is convenient when comparing behaviors at different speeds.

### Estimation of intersegmental coupling strengths and other parameters

Stepping sequences from unperturbed runs were used to fit a stochastic version of the linearized six-oscillator model (Eqn 5) introduced above. We considered trials in which the animal traversed the tunnel at a relatively constant speed (±20%) for 120–160 steps, a number found sufficient for successful model fitting in preliminary studies using simulated sequences of different lengths.

For each trial, we constructed a sequence of leg indices *k_1_,k_2_*,…,*k_m_*ϵ{1,2,…,6} indicating the order of touchdown events, and corresponding sequences *t*_1_≤*t*_2_≤…≤*t_m_* of touchdown times and *d*_1_≤*d*_2_≤…≤*d_m_* of cycle indices, with *d*_1_=0. These allow us to relate the data to oscillator phases by assuming that θ_*k_j_*_(*t_j_*)≈*_d_j__* for each *j*. Specifically, we set *d_j_*−θ_*k_j_*(*t_j_*)_=σ_m_η*_j_*, where the _η_*_j_* are independent normally distributed random variables with mean 0 and variance 1 and σ_m_ is the measurement noise, assumed identical for all oscillators. To account for variability in the locomotive cycle, we modify the linearized system (Eqn 5) by adding noise to each oscillator:
(8)
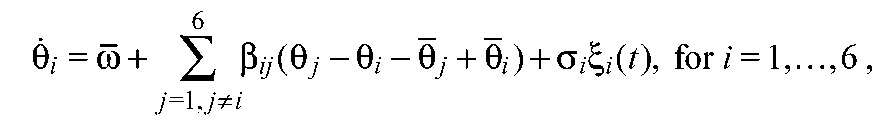

where ξ*_i_*(*t*) are independent Gaussian processes with intrinsic levels σ*_i_*, that are also independent from measurement noise. The number of coupling strengths α*_i,j_* can reach 14, depending on network architecture, and our model contains 12 additional parameters: 
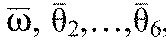
, σ_1_,…,σ_6_.

Parameters were estimated using a maximum-likelihood method (Harvey, 1990) to fit the linear stochastic system (Eqn 8). Given a vector **λ** of model parameters, a function *L*(**λ**) describing the likelihood of the data was computed. Parameter estimates for each trial were optimized by finding the λ^*^ that maximized log *L*(**λ**), using a quasi-Newton method (Dennis and Schnabel, 1983) as implemented in Matlab's fminunc function. The initial estimate of 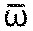 was calculated as the mean touchdown frequency and other parameters were randomly assigned. To approximate standard errors of parameter estimates, we computed the inverse of the Hessian matrix of −log *L*(**λ**) at λ^*^ and took the square roots of its diagonal elements Harvey (Harvey, 1990). Details are given in the Appendix. A similar method was previously used to fit pairs of nonlinear oscillators (Kiemel and Cohen, 1998; Kiemel et al., 2003; Fuchs et al., 2011).

### Simulation of recovery from perturbations

We simulated a stochastic version of the nonlinear phase oscillator (Eqns 3, 4):
(9)
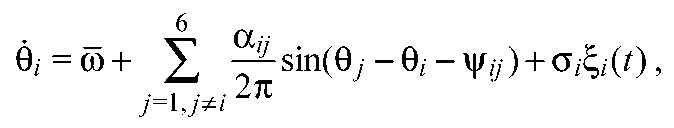

using parameters 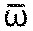, α*_i,j_*, σ*_i_* and 
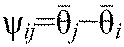
, estimated from the noisy touchdown data as described above. Thus, for the periodic phase-locked solution that best approximates the data, we have α*_i,j_*= β*_i,j_* cf. the phase-oscillator model described above. To simulate behaviors outside the fitted ranges, we varied parameters while maintaining ratios similar to estimates from experiments (Figs 6 and 8). Simulations were done by the Euler–Maruyama method (Higham, 2001), and recovery times computed as for the experiments.

Although the magnetic field remains constant through its duration *T*_mag_, its influence on the moving leg most likely varied. To account for this, we tested two specific perturbations: a rectangular step of constant magnitude −*p*_1_ and duration *T*_mag_, and an exponentially decaying function −*p*_2_exp(−κ*t*/*T*_mag_) that falls to a negligible value at *t*=*T*_mag_. Values *p*_1_, *p*_2_ and κ were chosen such that the integrated functions are equal and therefore produce similar net phase changes. Specifically, we added a function p(*t*−*t*_0_) to the phase equation for the perturbed leg in Eqn 9:
(10)
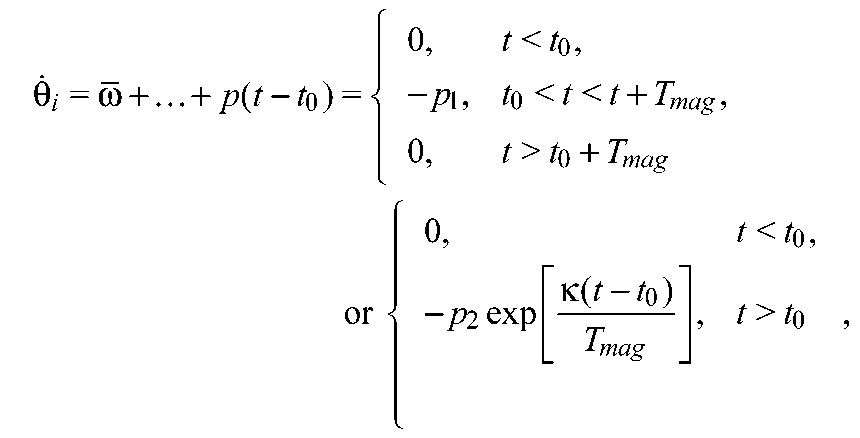

where *t*_0_ denotes perturbation onset. For all simulations we set *T*_mag_=50 ms to match the pulse duration in the experiment (as above) and chose *p*_1_=3, *p*_2_=30 and κ=10 to produce effects of similar magnitudes to those observed.

## Supplementary Material

Supplementary Material

## APPENDIX

### Details of perturbation experiments

#### Validation that magnets alone do not influence kinematics

To ensure that the magnet's weight and attachment to a single leg do not influence body or leg kinematics, we computed body yaw angles (body axis direction relative to the tunnel) and phase differences between leg pairs in trials with a magnet glued to a R1, R2 or R3 leg and the magnetic field inactive. Comparing these with analogous yaw angle and phase differences from trials without magnets, no differences were observed (*P*=0.45, two-way analysis of circular data), suggesting that the magnet alone does not influence leg phases or kinematics.

#### The influence of magnetic perturbations

As noted in the main text, perturbations to single legs had no significant effects on the speed or path of the animals' mass centers. Their influence on path length and cycle duration of the perturbed leg was relatively short (less than one cycle in all but one case). The relatively long magnetic pulse duration of 50 ms was chosen to provide sufficient force to produce observable effects on leg phases. It corresponds to a stance or swing period at 10 Hz and to a full step at 20 Hz, but we note that effects were greatest early in the period, as indicated by the simulations with decaying vs constant magnitude pulses shown in Fig. 6A.

Effects depended on the phase within the cycle when the perturbation began, as shown in supplementary material Fig. S1B. In general, we observed that perturbations applied to legs at anterior positions in swing or stance resulted in shortened path lengths and cycle durations, whereas perturbations arriving at legs in posterior positions had either no effect or caused slight cycle lengthening. Supplementary material Fig. S1A shows an example of the relation between a leg's position along the normalized *x*-(body) axis and its resultant phase. In supplementary material Fig. S1B we plot average values of path change (blue dots) and cycle duration change (green squares) relative to the *x*-position of the perturbed leg. Path change was computed as change in the perimeter of tarsi locations on the *x*- and *y*-axes in comparison with the average path length before perturbation, whereas cycle changes denote difference in cycle duration compared with the average pre-perturbation duration. Values were obtained from all tested animals perturbed at front, middle or hind legs. Similar information is encoded in the self-effPRCs (see Fig. 2A1–A3 and Discussion); in both cases, positive values correspond to step shortening.

### Details of the phase oscillator model, normalization of recovery times and parameter estimation

#### Derivation of the linearized phase oscillator

Here, we provide details of the linearization of the phase oscillator Eqn 3 in the main text. To linearize around the phase-locked solution , with common frequency  and constant phase differences  we expand the coupling functions *H_ij_* in Taylor series to obtain:

(A1)

Here,  is the derivative of *H_ij_* evaluated at  denotes terms of quadratic order in the phase differences that include second and higher order derivatives, and we use the fact that  in the second step. Finally, we set  and neglect the  terms to obtain Eqn 5 in the main text. The fact that θ_*i*_ appears in each of the functions *H_ij_*(θ_*j*_–θ_*i*_) leads to the expression , resulting in the neutral eigenvalue of ***B*** with eigenvector (1,…,1)^T^, as noted in the main text.

#### Normalization of recovery times

Here, we derive the expression for normalized mean recovery times. As described in main text, let *i* denote the perturbed leg, *j* the other leg of the pair, θ_*i,k*_ the phase at perturbation onset in trial *k*, and *n_i,j,k_* the number of cycles to recovery. Recall that *Z_i_*(θ_*i,k*_), the effPRC value at perturbation onset in trial *k*, is a measure of self-perturbation strength. The normalized perturbation strength for trial *k* is defined as:

(A2)

The number of cycles to recovery *n_i,j,k_* measured as described in main text is then divided by  to account for perturbation strength, and the resulting values averaged over all *N* trials for a given insect with the magnet on leg *i*. This yields the normalized mean recovery time for the leg pair *i,j*, Eqn 7 in the main text, via the following step:

(A3)

Fig. 4A in the main text plots data points in the form [*n_i,j,k_*,|*Z_i_*(θ*_i,k_*)|] to illustrate the linear trend in recovery time vs perturbation strength that is implicit in our normalization.

#### The likelihood computation

The likelihood function used in parameter estimation was computed using an approach previously used to fit parameters in a nonlinear model of two coupled phase oscillators (Kiemel and Cohen, 1998; Kiemel et al., 2003; Fuchs et al., 2011). The present computations require the fitting of six phase oscillators. However, computations are simplified because Eqn 8 in the main text is linear (in the narrow sense that the noise levels σ*_i_* are constant) (Arnold, 1974), so that all probability densities are Gaussian and thus characterized by their means and covariance matrices (see below).

We model variability in the free running data by assuming that the leg indices *k_j_*ϵ{1,2,…,6} and touchdown times *t_j_*, defined in main text, are fixed. We take as random variables the phases  measured at the fixed times *t_j_*, where . Here the η*_j_* are independent normally distributed random variables with mean 0 and variance 1 and σ*_m_* is the measurement noise level, as defined in main text. This extends the assumption from the main text that θ*_j_*=0 at touchdown of leg *j* by allowing for noise in the measurement of touchdown times and by tracking absolute phases θ*_j_*ϵ[0,∞] instead of θ*_j_*ϵ[0,1] modulo 1, so that absolute phases increase from one step to the next as described by Eqn 3.

The phases  are in principle continuous random variables but since they derive from successive leg touchdown data in practice they are non-decreasing sequences of integers. Typical opening entries of the sequence  might therefore be {0,0,0,0,0,0,1,1,1…}, logging the touchdown phases of the first three tripods of legs at times *t*_1_,…,*t*_9_, with identifying leg indices such as θ_2_(*t*_1_),θ_6_(*t*_2_),θ_4_(*t*_3_),θ_3_(*t*_4_),θ_5_(*t*_5_),θ_1_(*t*_6_),θ_6_(*t*_7_),θ_2_(*t*_8_),θ_4_(*t*_9_),… Referring to Fig. 5A in the main text for the numbering convention for right (R*j*) and left (L*j*) legs, this indicates that legs 2=R2, 6=L3 and 4=L1 of one tripod touch down in that order, followed by legs 3=R3, 5=L2, and 1=R1 of the other tripod to complete the first step. In the second step legs 6=L3, 2=R2 and 4=L1 follow, each having completed one full cycle, etc. If one or more legs fail to touch down during the *j*-th step fewer than six entries *j,j*,… appear.

Noting that the random variables  implicitly depend on the vector **λ** of model parameters, we define the likelihood function L(**λ**) as the conditional probability density of (), given that , evaluated at . We condition on the first measurement, because the equilibrium probability density of  is not well defined because of neutral eigenvalue of the linear system (Eqn 8). Indeed, recalling the definition of the deterministic system (Eqn 5), this stochastic differential equation (SDE) can be written as

(A4)

where ***S*** is a diagonal matrix with components *S_ii_*=σ*_i_* and the matrix ***B*** has a zero eigenvalue with eigenvector (1,…,1)^T^, as noted in main text. We shall use vector form (Eqn A4) below.

To compute *L*(λ) for a given λ, let the vector ***m***={*k*_1_,…, *k_j_*;*t*_1_,…,*t_j_*;*d*_1_,…,*d_j_*} denote measurements up to and including the *j*-th touchdown time and *p_j_*(*d_j_*) be the conditional probability density of  given the data ***m***_*j*−1_ evaluated at . The likelihood can then be written as the product of these conditional probability densities:

(A5)

Because the SDE (Eqn A4) is linear and measurement errors are Gaussian, each conditional phase density *p_j_* is also Gaussian, and thus is completely characterized by its conditional expected value  and conditional variance . Taking the log of Eqn A5 we therefore find that:

(A6)

Let  and  be the conditional vector expected value and conditional covariance matrix, respectively, of θ(*t_j_*) given ***m****_j_*_−1_. (Here the superscripts *j* identify leg touchdowns, because we will use subscripts to denote components of μ^(*j*)^ and Σ^(*j*)^.) Our measurement model  implies that:

(A7)

and given  and Cov[θ(*t*)|***m***], we can compute μ^(*j*)^ and Σ^(*j*)^ for *j*=2,…,*m* using a continuous-time Kalman filter with discrete measurements (Zhang et al., 2005). Eqns A6 and A7 can then be used to compute the likelihood *L*(λ). Therefore, it only remains to describe how to compute  and Cov[θ(*t*_1_)|***m***_1_].

To compute , we assume that the distribution of relative phases is at equilibrium at the time of the first touchdown, so that each component . To compute Cov[θ(*t*_1_)|***m***_1_], let ***M*** be the 5-by-6 matrix such that  is the vector of relative phases θ*_i_*−θ_*k*1_ for *i*=1,…*k*_1_−1, *k*_1_+1,…,6. Also let ***M***^+^ be the 6-by-5 matrix obtained by taking the 5-by-5 identity matrix ***I***_5_ and inserting a row of zeros at row index *k*_1_.

Noting that ***M***^+^ is a right pseudo-inverse of ***M***^+:^***MM***^+^ = ***I***_5_ [see section 3.4 in Strang (Strang, 1976)], we may transform Eqn A4 above to:

(A8)

Then, based on the theory of linear stochastic differential equations (Arnold, 1974), the equilibrium covariance matrix  of  is the solution of the linear matrix equation:

(A9)

From the definition of ***M***^+^ above,  is the vector of relative phases θ*_i_*−θ*_k_*_1_ and the equilibrium covariance matrix of ϕ is . For each component of θ we can write θ*_i_*(*t*_1_)=[θ*_i_*(*t*_1_)−θ*_k_*_1_(*t*_1_)]+ θ*_k_*_1_(*t*_1_)= ϕ*_i_*(*t*_1_)+σ*_m_*η_1_. By assumption, the phase dynamics and measurement noise η_1_ are independent, so that:

(A10)

where ***J***_6_ is the 6-by-6 matrix of ones and we have used the fact that the first touchdown provides no information about the relative phases ϕ(*t*_1_) or measurement noise σ*_m_*η_1_. This starts our Kalman filter process.

### Details of experimental data fitting and analysis

#### Intersegmental coupling parameters

As described in the main text, we used the stochastic version Eqn 8 of the phase oscillator model to estimate intersegmental connectivity strengths. Including only nearest-neighbor connections between all pairs of adjacent units (see Fig. 5A), the model potentially has 14 different coupling strengths α*_ij_*. To determine how many of these are needed to capture the dynamics of the running sequences, and to investigate (approximate) symmetries, we started by fitting these 14 independent coupling parameters and then successively considered the following cases with increasing degrees of symmetry: 11 parameters – assuming symmetry in contralateral couplings α_14_=α_41_, α_25_=α_52_, α_36_=α_63_; nine parameters – partial bilateral symmetry, assuming the above and α_12_=α_45_, α_21_=α_54_; seven parameters – full bilateral symmetry, assuming the above and α_23_=α_56_, α_32_=α_65_; six parameters – partial rostro-caudal symmetry, assuming the above and α_14_=α_36_; four parameters – full rostro-caudal symmetry, assuming the above and α_12_=α_32_, α_21_=α_23_; three parameters – assuming the above and α_12=_α_14_; two parameters – one for all ipsilateral and one for all contralateral connections; one parameter – all coupling strengths equal.

An alternative 11-parameter connection architecture with diagonal and contralateral connections was also investigated. This included pairwise connections between the three units in each tripod (α_15_, α_51_, α_24_, α_42_, α_26_, α_62_, α_35_, α_53_) and symmetric nearest-neighbor contralateral coupling α_14_=α_41_, α_25_=α_52_ and α_36_=α_63_.

#### Determining number of coupling parameters according to information criteria

The qualities of fits for different coupling configurations were assessed using Akaike and Bayesian Information Criteria (AIC and BIC) (Akaike, 1974; Akaike, 1981). These metrics evaluate goodness of fit while penalizing extra parameters. We computed AIC and BIC scores for each connection architecture and fitted parameter values using the log likelihood of how well it recovers the experimental data to determine the optimal number of independent coupling parameters. We also compared the nearest-neighbor architectures to the diagonal and contralateral architecture described above.

Supplementary material Table S1 lists BIC values of the different tested configurations for each preparation. Lower BIC scores indicate better fits and the blue cells in each line identify the optimal configuration for that preparation. As can be seen, no more than seven coupling parameters were needed to sufficiently recover the experimental data for all preparations. AIC scores (not shown) revealed a similar result.

#### Absolute values of coupling parameters

As noted in main text, absolute values of estimated coupling and noise parameters vary substantially among preparations. In supplementary material Table S2 the estimated frequency, coupling strengths and the square root of sum of squares of noise estimates:

(A11)

are given for each preparation (see supplementary material Table S3 for individual σ*_j_* values). As can be seen from these values and in supplementary material Fig. S2, the average coupling strengths:

(A12)

co-vary with estimated noise levels (*P*<0.05, linear regression analysis), suggesting that differences in optimal solutions found may be attributed to differences among the seven preparations in noise levels and running path variability.

Supplementary material Table S3 lists the estimated preferred phase differences  between the first and other five legs estimated for the different preparations. As noted in the main text, these values implicitly determine all preferred phase differences. Individual noise strengths σ*_i_* and measurement noise σ*_m_* are also listed.

#### Distance from the balanced subspace

Using the seven-parameter architecture, the conditions under which inputs entering each leg are balanced are characterized by:

(A13)

as described in main text. Eqn A13 constrains two of the seven independent coupling parameters and thereby defines a five-dimensional balanced subspace *B*. We define the distance to balance for each preparation as its Euclidian distance from *B* by taking the vector of coupling strengths  and computing its normal projection onto *B*:

(A14)

where ***A*** is a 7-by-5 matrix whose columns span *B*. Specifically, we chose:

(A15)

The distance of ***c*** from *B* is then ***||c***−***Pc||***. Normalized distances from *B* are computed as ratios of ***||c***−***Pc||*** to the magnitude of the component of ***c*** parallel to *B*:

(A16)

where ***I***_7_ is the 7-by-7 identity matrix and ***||***·***||*** denotes the Euclidean norm.
