# Supplementary material for: Intersegmental coupling and recovery from perturbations in freely running cockroaches
Source: J Exp Biol. 2015 Jan 15;218(2):285–97. doi: 10.1242/jeb.112805 (PMC4302167; doi:10.1242/jeb.112805)
Supplement: Supplementary Material [file supp_218_2_285__index.html]

Intersegmental coupling and recovery from perturbations in freely running cockroaches — Supplementary Material 

# Intersegmental coupling and recovery from perturbations in freely running cockroaches

## JEB112805 Supplementary Material

**Files in this Data Supplement:**

- **Supplementary Material**
