# Supplementary material for: Intersegmental coupling and recovery from perturbations in freely running cockroaches
Source: J Exp Biol. 2015 Jan 15;218(2):285–97. doi: 10.1242/jeb.112805 (PMC4302167; doi:10.1242/jeb.112805)
Supplement: Supplementary Material [file supp_218.2.285_JEB112805.pdf]

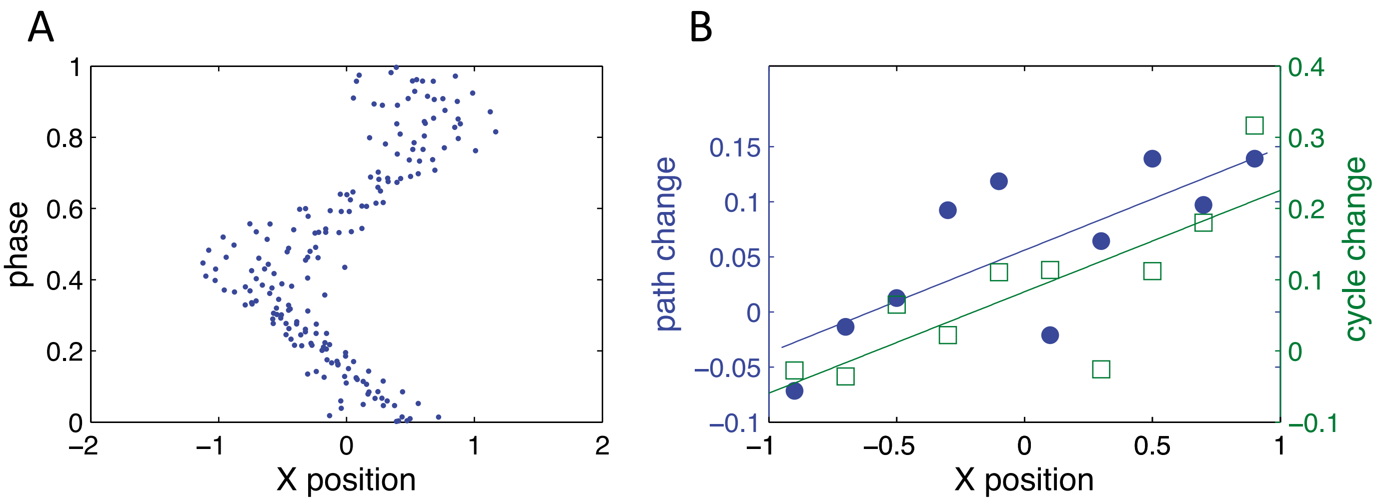

Fig. S1. (A) An example of mapping normalized tarsi positions on the *x*-axis to phases over the stance/swing cycle. (B) Effects of perturbations on tarsus path length (*x*-position, blue dots) and cycle duration (open green squares) of perturbed leg as functions of normalized *x*-position at perturbation onset. Regression lines show stride lengths shortening for perturbations at anterior leg positions.

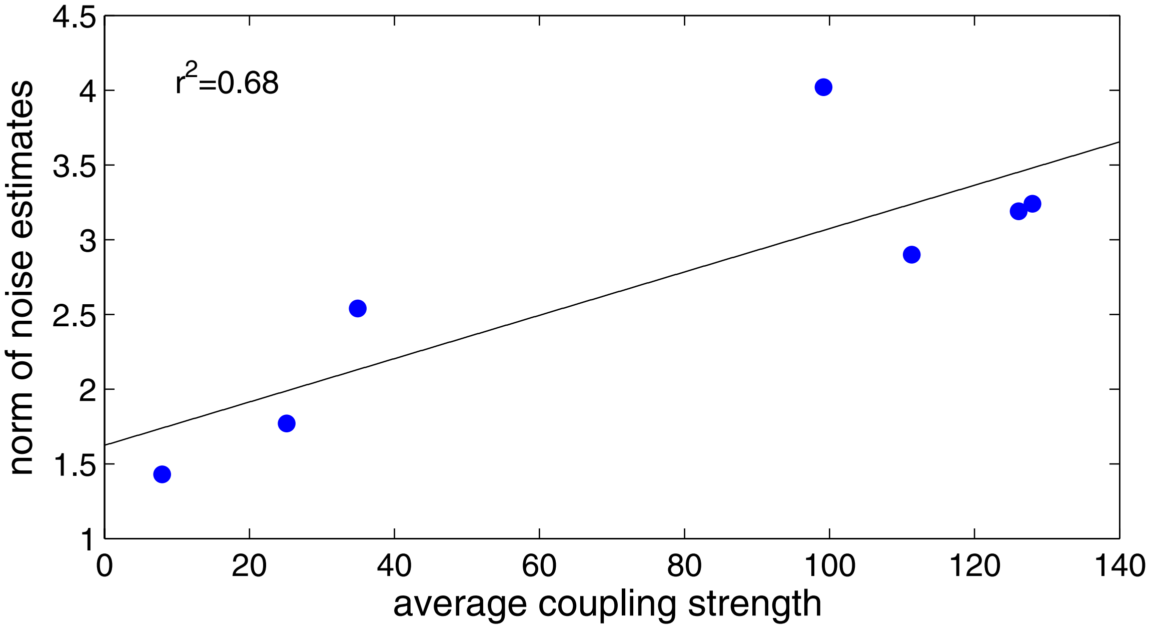

Fig. S2. Average absolute coupling strengths $\frac{1}{7}\sum_{i=1}^{7} c_j$ for each preparation plotted against the norm of noise estimates $\bar{s}$, showing a significant correlation (P<0.05).

Table S1. Bayesian Information Criteria for different model architectures

Number of coupling parameters									
14 par	11 par	9 par	7 par	6 par	4 par	3 par	2 par	1 par	Diagonal
-409.35	-402.96	-422.3	-399.47	-406.42	-433.06	-426.15	-354.14	-384.57	-401.81
-373.4	-371.22	-379.98	-397.37	-401.56	-380.51	-369.62	-353.56	-378.6	-346.14
-617.51	-602.27	-610.17	-632.34	-624.61	-628.27	-581.22	-492.97	-378.6	-585.16
-922.85	-942.26	-947.19	-951.4	-954.77	-959.77	-963.31	-870.71	-930.18	-840.61
-569.88	-575.37	-583.66	-590.37	-593.38	-623.7	-592.79	-521.44	-586.66	-486.67
-816.74	-875.5	-883.6	-880.88	-897.46	-884.88	-888.51	-726.09	-842.72	-617.16
-611.32	-614.18	-622.59	-630.69	-635.56	-642.87	-646.56	-588.59	-634.27	-460.78

Preparations are listed in the same order as in Tables S2 and S3 and in Fig. 5B of the main text.
Lowest BIC scores are marked in blue.

Table S2. Absolute coupling strengths c_j and average intrinsic noise levels $\bar{\sigma} = \sqrt{\sum_{1=1}^{6} \sigma_i^2}$

Estimated parameters								
Frequency	c_1	c_2	c_3	c_4	c_5	c_6	c_7	$\bar{\sigma}$
4.17	6.61	4.75	8.93	6.5	9.65	14.12	5.16	1.43
4.18	33.52	27.8	80.74	19.02	30.26	32.88	20.42	2.54
11.46	222.14	101.9	151.01	89.05	116.89	123.26	78.31	3.19
14.26	166.58	66.83	205.01	107.23	135.5	113.34	101.43	3.24
14.59	20.06	12.67	22.68	23.58	26.75	50.49	19.73	1.77
15.61	184.17	93.09	194.92	57.44	52.16	54.52	57.92	4.02
18.28	97.11	20.92	192.37	113.29	91.74	158.92	105.12	2.90

Table S3. Preferred phase differences $\psi_{ij} \in [0,1]$ (modulo 1), individual noise magnitude estimates σ_i and measurement noise estimates σ_m

frequency	ψ_{21}	ψ_{31}	ψ_{41}	ψ_{51}	ψ_{61}	σ_1	σ_2	σ_3	σ_4	σ_5	σ_6	σ_m
4.17	0.467	0.963	0.494	0.976	0.468	0.71	0.23	0.65	0.77	0.63	0.24	0.08
4.18	0.467	0.926	0.434	0.977	0.416	1.20	0.40	0.69	1.89	0.24	0.87	0.05
11.46	0.483	0.940	0.425	0.001	0.403	1.31	0.71	1.02	0.98	1.94	1.48	0.01
14.26	0.559	0.067	0.503	0.057	0.548	1.32	1.18	1.51	0.89	1.80	0.90	0.06
14.59	0.550	0.051	0.478	0.035	0.536	0.82	0.06	0.87	0.93	0.22	0.88	0.01
15.61	0.615	0.058	0.600	0.134	0.576	0.48	2.74	1.30	0.60	2.45	0.59	0.10
18.28	0.580	0.119	0.460	0.098	0.618	0.92	1.38	1.25	1.50	1.02	0.90	0.07
